# The Emergence of Novel Variants of the Porcine Epidemic Diarrhea Virus Spike Gene from 2011 to 2023

**DOI:** 10.1155/2024/2876278

**Published:** 2024-07-16

**Authors:** Lin Zhang, Jian-Bo Liu, Hui-Zhen Liu, Yue-Xiao Lian, Yao-Wei Huang, Feng Cong

**Affiliations:** ^1^ College of Veterinary Medicine South China Agricultural University, Guangzhou 510642, China; ^2^ Guangdong Laboratory Animals Monitoring Institute, Guangzhou 510663, China; ^3^ State Key Laboratory of Respiratory Disease CAS Key Laboratory of Regenerative Biology Guangdong Provincial Key Laboratory of Stem Cell and Regenerative Medicine Guangdong Provincial Key Laboratory of Biocomputing Guangzhou Institutes of Biomedicine and Health Chinese Academy of Sciences, Guangzhou 510530, China; ^4^ Guangdong Laboratory for Lingnan Modern Agriculture, Guangzhou 510642, China

## Abstract

The porcine epidemic diarrhea virus (PEDV) has caused severe economic losses in the pig industry. Since its discovery, the virus has spread in pig herds for more than 50 years. Many new features have been found in the PEDV spike genes. In this study, 123 representative S genes were used to analyze their characteristics across strains. Phylogenetic analysis revealed that PEDV can be divided into nine groups: G1a, G1b, G1c, G1d, G2a, G2b, G2c, G2d, and G3. In addition, 21 different lengths of the S gene were found. Analysis of the amino acid insertion and deletion sites revealed that most deletions and insertions occurred in the loops of the spike quaternary structure, primarily in the D0 and N-terminal domains (NTDs). According to the above results, PEDV has undergone considerable evolution, possibly under the immune pressure of vaccination. These results are highly important for understanding the current epidemic situation of PEDV.

## 1. Introduction

Porcine epidemic diarrhea virus (PEDV) is a coronavirus that can infect pigs of all ages; it mainly infects neonatal piglets [1] and has spread among pig herds for more than 50 years since it was first discovered in 1971 [2]. The main symptoms of piglets infected by PEDV are acute diarrhea and vomiting, and piglets have a high rate of mortality [3]. Until 2010, PEDV strains consisted of only one genotype, G1 (CV777, which is the representative strain with a 4152-nt S gene). However, since 2010, a new genotype named G2 (LNCT2 as the representative strain with a 4161-nt S gene) has emerged with an S-gene length 9 nt longer than that of G1 strains [4, 5], with the mutated nucleotides being neglected. The G2 strain emerged in the USA in 2013 and has caused great economic loss; moreover, until recently, no G1 strains were detected in pig herds in the USA [6].

The S gene of coronaviruses encodes the S protein, which forms a homotrimeric spike complex. Coronavirus spikes are Class I viral fusion proteins [7]. These spikes are located on the surface of coronaviruses and play important roles in target cell entry [7, 8]. Additionally, the spike protein can induce a humoral immune response. To date, many different variations in the S gene have been found. Therefore, in this study, we focused on the nucleotides and amino acids of the S gene and analyzed them to provide new insights into strategies for controlling this virus.

## 2. Materials and Methods

### 2.1. PEDV Sequences from GenBank

Representative PEDV S-gene sequences from 2011 to 2023 were downloaded from GenBank and are listed in *Supplementary Table 1*.

### 2.2. MEGA and DNASTAR

MEGA 11 software (https://www.megasoftware.net/) was used to construct a phylogenetic tree with default parameters and manual adjustments. MegAlign in DNASTAR was used to analyze the amino acid sequence of PEDV as well as its nucleotide homology.

### 2.3. Amino Acid Insertion and Deletion in the Spike Protein Structure

The PEDV spike structure (Protein Data Bank (PDB): 7Y6V) was used as a model to show the amino acid insertions and deletions via Chimera (1.17.3) software (downloaded from UCSF Chimera).

## 3. Results

### 3.1. Spike Genes of Different Lengths

As shown in *Supplementary Table 1*, the PEDV S genes were of different lengths. The shortest S gene was 3,570 nt (with the D0 domain missing compared to that of KR078299), while the second shortest gene was 3,984 nt (with 169 nt at the N-terminus missing compared to that of AF353511). The longest S gene was 4,182 nt in length. In contrast to the two shortest S genes (3,570 and 3,984 nt), the lengths of the PEDV S genes in GenBank range between 4,125 and 4,182 nt, increasing with the addition of multiples of three nucleotides. Each corresponding length was represented by at least one strain, except for 4,137 nt, which has not been found in any strain to date (4125 + 3 × *N*, 0 ≤ *N* ≤ 19, *N* ≠ 4, where *N* is an integer).

### 3.2. Analysis of the Genetic Variation of the Spike Genes

**Figure 1:**
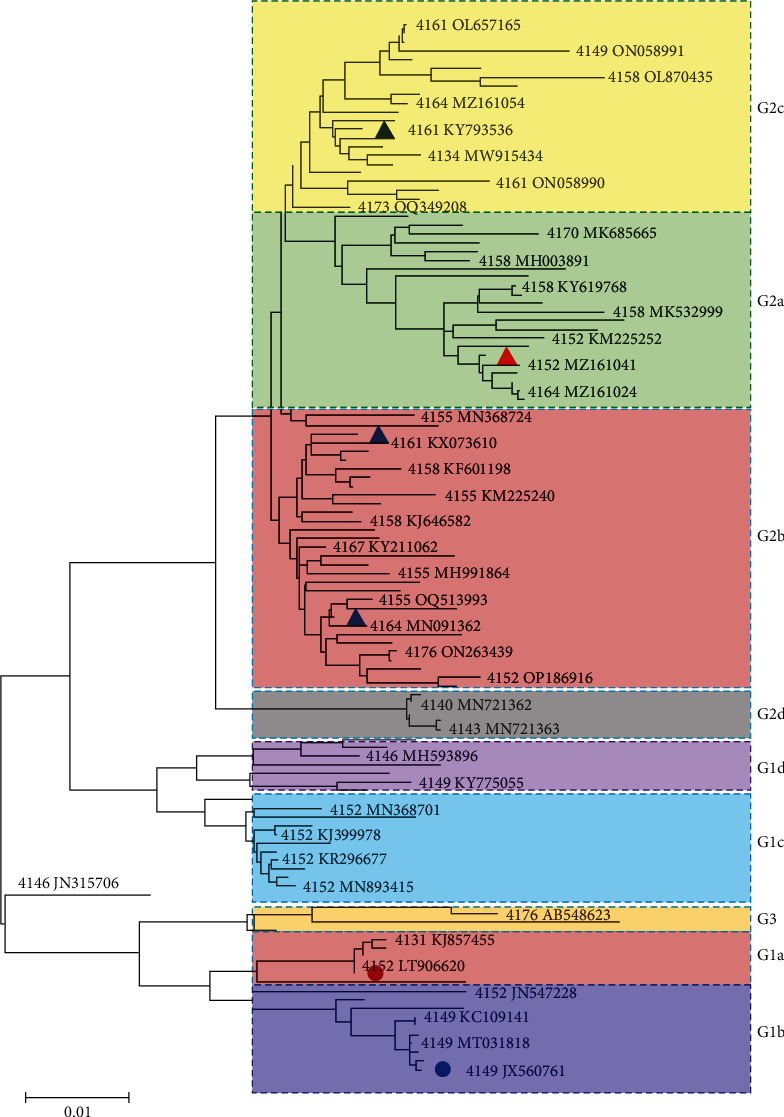
Phylogenetic analysis of PEDV based on the S gene. The gap opening penalty was changed to 10 from the default 15 using the maximum likelihood method. The labeled circle sequences are representative of G1 sequences, while the triangle-labeled sequences are representative of G2 sequences. G1a AF353511, G1b JX560761 (4,149), G2a JX647847, G2b KF468753, and G2c KY793536 are cited from Wen et al. [9].

To understand the relationships between S genes of different lengths, the S genes (except those with a length of 3,570 or 3,984 nt) were aligned and analyzed via MEGA 11 software. As shown in Figure 1, the PEDV S-gene sequences can be divided into nine subgroups: G1a, G1b, G1c, G1d, G2a, G2b, G2c, G2d, and G3. The classical strain CV777 (AF353511) was in the G1 genotype (G1a), while LNCT2 was in the G2 genotype (G2b). The G1c, G1d, G2d, and G3 genotypes were divided for the first time in this study. In addition, a new branch with just one sequence (JN315706) emerged in the phylogenetic tree.

## 4. The Main Differences in Spikes from Viruses of the Same Genotype

By analyzing the S gene from viruses of the same genotype, we obtained many new findings (*Supplementary Tables 2* and *3*). As shown in *Supplementary Table 2*, in G1a strains, there are three different S-gene lengths: 4,131, 4,143, and 4,152 nt. Compared with that of AF353511 (CV777), the two 4,131 nt sequences (KJ857455 and LT897799) had a 21-nt deletion at the end of the S2-encoding region before the termination codon (TGA). The 4,143 nt sequence (OQ291158) also had a 21-nt deletion at the end of the S2-encoding region before the termination codon (TGA) but had a 12-nt insertion in the 331 site in the NTD-encoding sequence.

In G1b strains, there are three different S-gene lengths: 4,146, 4,149, and 4,152 nt. All the sequences were compared with AF353511. All the sequences (except those of JN547228 and MW145531) in the G1b strains had 3-nt deletions at 455–457 nt, which is a special characteristic of G1b sequences. In addition, the MN315264 (4,146 nt) sequence has a 3-nt deletion at the 2,323–2,325 site.

In the G1c strains, three S genes with lengths of 4,143, 4,146, and 4,149 nt were detected. All the sequences were compared with that of AF353511. All the sequences (except for those of MK820042 and MZ16108) in the G1c strains had 3-nt deletions at 3,578–3,580 nt, which is a special characteristic of G1c sequences. In addition, the KX982564 (4,143 nt) sequence has a 3-nt deletion at 329–331 nt and a 3-nt deletion at 401–403 nt. The MH593896 (4,146 nt) sequence has a 3-nt deletion at the 400–402 position. The MK820042 and MZ161081 (4,149 nt) sequences had 3-nt deletions at residues 400–402.

In G1d strains, there are two different S-gene lengths, 4,149 and 4,152 nt. Compared with AF353511, the KP399615 (4,149) sequence has a 3-nt deletion between 1,068 and 1,108 nt.

In G2a strains, there are seven different S-gene lengths: 4,152, 4,155, 4,158, 4,164, 4,167, 4,173, and 4,182 nt. All the sequences were compared with that of KT323980. All the sequences in the G2a strains (except MZ090589) had 3-nt deletions at 3,589–3,591 nt; this is a special characteristic of G2a sequences. In addition, there are many specific differences. The KM225252 (4,152 nt) sequence has a 6-nt deletion at 430–435 nt. The MH991856 and MZ161041 (4,152 nt) sequences had 6-nt deletions at 164–169 nt. The MW915437 (4,152 nt) sequence has a 6-nt deletion at nucleotides 182–187. The KR941552 (4,155 nt) sequence has a 3-nt deletion at 2,237–2,239 nt. KF601199 has a 3-nt deletion at 3,489–3,491 nt, one nucleobase insertion at the 1,190 site and one nucleobase deletion at the 1,214 site. The MZ161024 (4,164 nt) sequence has a 6-nt insertion at the 3,812 site. The KP870139 (4,167 nt) sequence has a 9-nt insertion at the 4,139 site. The MZ161023 (4,167 nt) sequence has a 9-nt insertion at the 3,812 site. The JQ638918 (4,170 nt) sequence has a 12-nt insertion at the 693 site. The MK685665 (4,170 nt) sequence has a 12-nt insertion at the 1,126 site. The MZ161059 (4,170 nt) sequence has a 12-nt insertion at the 3,812 site. The MZ090589 (4,173 nt) sequence has a 12-nt insertion at the 700 site. The KX982576 (4,182 nt) sequence has a 24-nt insertion at the 1,149 site.

In G2b strains, there are 11 different S-gene lengths: 4,128, 4,149, 4,152, 4,155, 4,158, 4,161, 4,164, 4,167, 4,170, 4,173, and 4,176 nt. All the sequences were compared with that of KT323980. The 4,128-nt sequence of MW915432 has a 6-nt deletion at the 164–169 site and a 27-nt deletion at the 3′ end of the S gene before the termination codon (TAA); the 4,149-nt sequence of MF038016 has a 12-nt deletion at the 342–353 site; the 4,152-nt sequence of ON263446 has a 9-nt deletion at the 1,127–1,135 site; the 4,155-nt sequence of KT313038 has a 6-nt deletion at 1,136–1,141 nt; the 4155-nt sequences of MH991864, OQ513993, KM225240, and KM225244 have a 6-nt deletion at the 164–169 site; the OP186916 (4,152 nt) sequence has a 6-nt deletion at 181–186 nt and a 3-nt deletion at 416–418 nt; the OP186873 (4,155 nt) sequence has a 6-nt deletion at 181–186 nt; and the KF601200 and KF601201 (4,161 nt) sequences have a 3-nt deletion at 1,317–1,319 nt. The ON263439 and ON263440 (4,176 nt) sequences have a 15-nt insertion at position 191. The KP399608 and KP399630 (4,176 nt) sequences have a 15-nt insertion at the 1,128 site; the ON263438 (4,164 nt) sequence has a 3-nt insertion at the 1,139 site; the MN091362 (4,164 nt) sequence has a 3-nt insertion at the 1,146 site; and the KY211062 and OQ349203 (4,167 nt) sequences have a 6-nt insertion at the 1,151 site. The KY828998 (4,170 nt) sequence has a 9-nt insertion at the 693 site. The MK507906 (4,173 nt) sequence has a 12-nt insertion at the 693 site. The MG132636 (4,170 nt) sequence has a 9-nt insertion at the 701 site.

In G2c strains, there are 12 different S-gene lengths: 4,125, 4,134, 4,149, 4,152, 4,155, 4,158, 4,161, 4,164, 4,173, 4,176, 4,179, and 4,182 nt. All the sequences were compared with that of KT323980. The KM406180 (4,125 nt) sequence has a 36-nt deletion at 4,048–4,083 nt. The MW915434 (4,134 nt) sequence has a 6-nt deletion at 164–169 and a 21-nt deletion upstream of the termination codon (TGA). OL870434 has a 9-nt deletion in the 2,972–3,027 region and a 3-nt deletion in the 3,942 and 4,071 region with three nucleobases at three sites. The ON058991 (4,149 nt) sequence has a 12-nt deletion in the 164–185 region. The MZ161083 (4,152 nt) sequence has a 9-nt deletion in the 164–185 region. The MN368724 (4,155 nt) sequence has a 6-nt deletion in the 177–182 region. The OL870435 (4,158 nt) sequence has a 3-nt deletion in the 3,589–3,591 region. The MW478768 (4164 nt) sequence has a 3-nt insertion at the 1,664 site. The MZ161054 (4,164 nt) sequence has a 3-nt insertion at the 2,311 site. The OL870433 (4,164 nt) sequence has a 6-nt insertion in the 940–970 region and a 3-nt deletion in the 3,587–3,588 region. The OQ718904 (4,164 nt) sequence has a 3-nt insertion at the 1,176 site. The OQ349208 (4,173 nt) sequence has a 12-nt insertion at the 694 site. The MZ161008 (4,176 nt) sequence has a 15-nt insertion at the 1,078 site. The KY211046 (4,179 nt) sequence has an 18-nt insertion at the 1,066 site. The MK111633 (4,182 nt) sequence has a 21-nt insertion at the 1,072 site.

In G2d strains, there are two different S-gene lengths, 4,140 and 4,143 nt. All the sequences were compared with that of KT323980. All the sequences had a 12-nt deletion at 175–186 nt, a 3-nt deletion at 209–211 nt, and a 3-nt deletion at 420–422 nt. In addition, for KP890336, MN721362, and MN721368, there was another 3-nt deletion at positions 417–419.

In the G3 strains, there were three different S-gene lengths, 4,152, 4,164, and 4,176 nt. All the sequences were compared with those of AF353511 and KT323980. Compared with those of KT323980, all the sequences (except MN617863) in the G3 strains had a 3-nt insertion at 192 nt, which is a special characteristic of G3 sequences. In addition, the AB548623 (4,176 nt) sequence has a 12-nt insertion at the 1,830 site. The MN617863 (4,152 nt) sequence has a 12-nt deletion at positions 167–191 and a 3-nt deletion at positions 416–418, with a 6-nt insertion at the 480 site. Compared with AF353511, MN617863 has no nucleotide deletions or insertions; the deletions and insertions in AB548622, KY619779, and AB548623 are more complex than those in KT323980. Additionally, compared with those of KT323980, the similarities of MN617863, AB548622, KY619779, and AB548623 were 96.02%, 94.04%, 94.57%, and 94.37%, respectively; however, compared with those of AF353511, the similarities of MN617863, AB548622, KY619779, and AB548623 were 97.23%, 93.76%, 94.34%, and 94.31%, respectively. However, the G3 subgroup is similar to the G1 subgroup.

### 4.1. Nucleotide Insertions and Deletions Mainly Appeared in the D0 Domain and NTD

**Figure 2:**
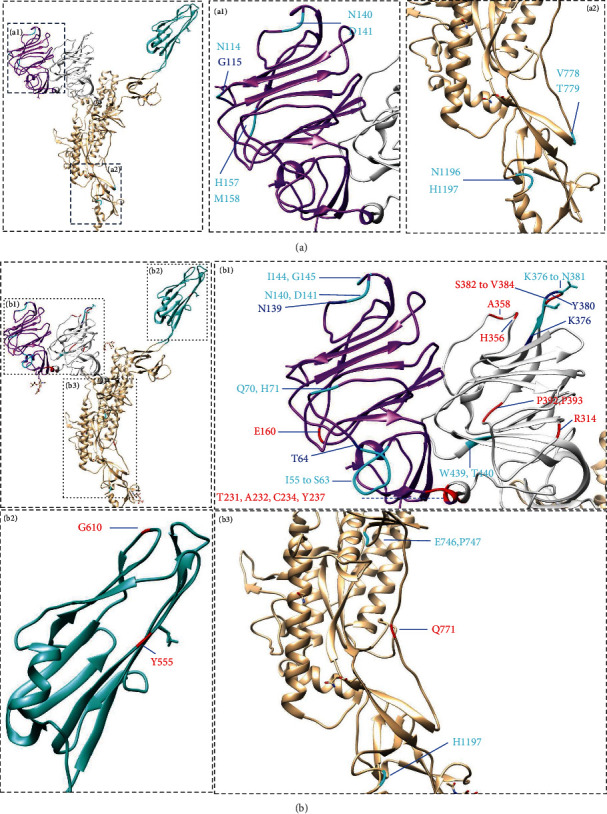
The amino acid deletion and insertion sites are shown in the spike structure. The 7Y6V structure was used as a model to show the deletion and insertion sites. Red indicates the insertion sites, cyan indicates the deletion sites, and blue indicates that the site can contain an insertion or a deletion. The purple color represents the D0 domain, the light gray color represents the NTD, the light sea green color represents the CTD, and the tawny color represents the S2 domain. (a) The deletions and insertions that appeared in the G1 subgroup strains are shown in (a1) and (a2). (b) The deletions and insertions that appeared in the G2 subgroup strains are shown in (b1), (b2), and (b3).

Based on the sequence comparison results, we identified the insertion and deletion sites in the structure deposited in the PDB. Red indicates the insertion sites, cyan indicates the deletion sites, and blue indicates that the site can be altered by either insertion or deletion. For the G1 subgroup, there is only one spike structure (PDB: 6U7K) [10] with several amino acid segments missing in the structure; therefore, we used the 7Y6V structure [11] as the model because it shows the regions involved in the amino acid deletion and insertions in the G1 subgroup. As shown in Figure 2(a), one site (G115) can be affected by deletion and insertion, three sites (N114, N140-D141, and H157-M158) can contain deletions in the D0 domain, two sites (V778-T779 and N1196-H1197) can contain deletions in the S2 domain, and no deletions or insertions appear in the NTD or C-terminal domain (CTD). For the G2 subgroup, four sites (T64, N139, K376, and Y380) had deletions and insertions, four sites (I55-S63, Q70-H71, N140-D141, and I144-G145) in the D0 domain had deletions, two sites (K376-N381 and W439-T440) in the NTD had deletions, and two sites (E746-P747 and H1197) in the S2 domain had deletions. Among the insertion and deletion sites, the D0 domain has two sites (T64 and N139), and the NTD has two sites (K376 and Y380). Among the insertion sites, the D0 domain has three sites (E160, T231-A232, and C234), the NTD has six sites (Y237, R314, H356, A358, S382-V384, and P392-P393), the CTD has two sites (Y555 and G610), and the S2 domain has one site (Q771). Almost all the deletion and insertion sites are located in the loop, and only one site (Y555) is located in the *β*-sheet.

### 4.2. Intragroup and Intergroup Homology Analyses

As shown in Tables 1 and 2, the intragroup sequence similarity was greater than 95.5%. According to the intergroup analysis, the sequences in the G1a subgroup were similar to those in the G1b subgroup, with 95.7%–97.6% similarity, which was greater than that among the other groups. For the G2b group, the sequences were similar to those in the G2c group, with a similarity of 96.2%–99.2%, which was greater than that among the other groups. For the G2a group, the sequences were similar to those in the G2d group, with a similarity of 95.2%–97.0%, which was greater than that among the other groups. For the G1d group, the sequences were similar to those in the G1c group, with 95.7%–97.8% similarity, which was greater than that among the other groups.

## 5. Discussion

PEDV is an important coronavirus that infects piglets. Since the virus first spread worldwide, it has caused significant economic losses every year. Regarding the control of these viruses, many vaccines based on the G1 (e.g., the CV777 vaccine; Harbin Weike Biotechnology Development Company) and G2 (e.g., AJ1102; Wuhan Keqian Biology Co., Ltd.) strains have been approved by governments and used by the pig industry. However, immunization with either of the two vaccine genotypes cannot completely protect against the virus. The reasons for incomplete protection by vaccines are complex and are not discussed here.

In the G1 and G2 groups, many strains exhibited amino acid deletions. The causes of these deletions are unknown. In a previous study, Liu et al. [5, 12] demonstrated that the neutralizing antibody 1B9 lost its virus-neutralizing ability when the LNCT2 strain lost five amino acids at the 55–60 aa site under immune pressure. Additionally, the PEDV GDU strain could escape neutralization by the mAbs 63, 72, and 23 after the P129L, F100L, and V638G mutations, respectively [13]. These results suggested that the amino acid deletions and mutations may have appeared under the pressure of antibodies generated in response to the vaccines.

Moreover, in SARS-CoV-2, most epitopes of neutralizing monoclonal antibodies are located in loop regions [14, 15]. Thus, we can hypothesize that the loop regions with high PEDV flexibility may be the binding sites of neutralizing antibodies, and under immune pressure, amino acids in these regions mutate for virus survival. However, there are many amino acid insertion sites in many different strains. These insertions appeared mainly in the G2 genotype strains (Figure 2), and only two insertion sites emerged in the G1 strains. In addition to the insertion sites shown in Figure 2, two domains readily acquired insertion mutations that are not shown in the structure of the PDB. One insertion site (3,811–3,812 nt) is near the tail of the exterior region and before the TM region, and the other insertion site (4,138–4,139) is downstream of the TM region and located in the intracellular domain. For the G1 group strains, only deletions appeared in the intracellular domain before the termination codon. However, how deletions or insertions in the intracellular domain of the S gene affect the virus requires further research.

To date, no research on PEDV has revealed immune pressure-induced amino acid insertions. The reasons for the amino acid insertion into the spike protein, as well as the functions of the mutated spike protein, need further investigation.

Mutations, especially deletions and insertions in the S protein, may alter the virulence and tissue tropism of coronaviruses [16, 17]. Coexisting variants might be associated with persistent PEDV infection on pig farms. Such reemergence and coinfection of the variants analyzed in this study indicate a new situation for PEDV, suggesting that this disease has become more complex in terms of viral isolation, pathogenesis, and epidemiology.

With respect to the sequences in the G1 group strains, the S gene was no longer than 4,152 nt. During the early stage of G2 emergence, we could easily judge whether the PEDV strain belonged to the G1 or G2 group by observing the S gene length; a length of 4,152 nt was considered indicative of the G1 group, while 4,161 nt was considered indicative of the G2 group. However, the situation is now more complex. During the period from 2011 to 2023, many new variant strains with different lengths of the S gene emerged. Based on the phylogenetic tree, 4,131, 4,143, 4,146, 4,149, and 4,152 nt long S genes emerged in G1a and G1b, while 4,128, 4,134, 4,149, 4,152, 4,155, 4,158, 4,161, 4,164, 4,167, 4,170, 4,173, 4,176, 4,179, and 4,182 nt emerged in the G2a, G2b, and G2c subgroups. Several genetic and phylogenetic analyses have led to the identification of a special group named the “S-INDEL strains,” which might include the G1c, G1d, G2d, or G3 subgroup [9]. Considering the different lengths of the S gene in the G1a, G1b, G2a, G2b, and G2c strains, the S-INDEL strains may not be appropriate.

In summary, this study divided PEDV strains into nine genotypes and revealed that the loop domains readily acquire deletions and insertions. Each group has special characteristics which provide new insights into the characteristics and control of other coronaviruses, such as SARS-CoV and SARS-CoV-2.

## Figures and Tables

**Table 1:** Analysis of the intragroup sequence identity of the spike genes.

Groups	Identity (intragroup)
G1a	96.9%–100%
G1b	96.4%–100%
G1c	97.5%–99.7%
G1d	96.5%–99.0%
G2a	96.2%–99.9%
G2b	97.1%–99.9%
G2c	96.0%–100%
G2d	99.5%–99.9%
G3	95.3%–98.8%

**Table 2:** Analysis of the intergroup sequence identity of the spike genes (%).

	G1b	G1c	G1d	G2a	G2b	G2c	G2d	G3
G1a	95.7–97.6	94.5–95.9	94.5–95.5	93.0–94.1	93.1–94.2	93.0 −94.3	93.9–95.7	93.2–97.2
G1b	—	94.8–97.4	94.6–96.4	92.8–95.0	92.9–95.3	93.0–95.4	93.8–95.0	94.1–96.9
G1c	—	—	95.7–97.8	94.0–96.0	95.2–96.8	94.7–96.7	95.0–95.8	93.0–95.9
G1d	—	—	—	93.9–96.5	94.3–96.3	94.4–96.6	94.5–95.5	92.3–95.5
G2a	—	—	—	—	95.7–98.9	94.9–98.6	95.2–97.0	93.4–96.6
G2b	—	—	—	—	—	96.2–99.2	96.0–97.5	93.5–96.7
G2c	—	—	—	—	—	—	95.7–97.2	93.2–96.7
G2d	—	—	—	—	—	—	—	93.7–96.4

## Data Availability

All the data generated in this study are presented within the manuscript and its Supplementary Materials files.

## Supplementary Materials

Supplementary 1 Table 1: information on the PEDV strains used in this study.

Supplementary 2 Table 2: deletions and insertions of the sequence in the G1 group compared with the AF353511 (CV777) sequence.

Supplementary 3 Table 3: deletions and insertions of the sequence in the G2 group and the G3 group compared with the KT323980 (LNCT2) sequence. (Additional supporting information can be found online in the Supplementary Materials section at the end of this article.)
