# Supplementary material for: The Emergence of Novel Variants of the Porcine Epidemic Diarrhea Virus Spike Gene from 2011 to 2023
Source: Transbound Emerg Dis. 2024 Jul 16;2024:2876278. doi: 10.1155/2024/2876278 (PMC12017180; doi:10.1155/2024/2876278)
Supplement: Supplementary 1 — Table 1: information on the PEDV strains used in this study. [file 2876278.f1.docx]

Table S1. Information on the PEDV strains used in this study

| S gene (nt) | GenBank No. | Name | Year | Country | Genotype |
| --- | --- | --- | --- | --- | --- |
| 3570 | KY499261 | TC-PC177 | 2017 | USA | null |
| 3984 | OM814174 | CN19 | 2022 | China | null |
| 4125 | KM406180 | DY1403 | 2014 | China | G2c |
| 4128 | MW915432 | YJH/2015 P170 | 2022 | China | G2b |
| 4131 | LT897799 | GER_L00901-V215_1978 | 2018 | Germany | G1a |
| 4131 | KJ857455 | SM98-5P | 2016 | Korea | G1a |
| 4134 | MW915434 | HY/2017 P150 | 2022 | China | G2c |
| 4140 | MN721368 | CH-SX-YL27 | 2019 | China | G2d |
| 4140 | KP890336 | CH/HNYF/14 | 2015 | China | G2d |
| 4140 | MN721362 | CH-SX-YL6567 | 2019 | China | G2d |
| 4143 | OQ291158 | Avac/PEDV/98 | 2023 | Vietnam | G1a |
| 4143 | KX982564 | VN232/HB/2013 | 2017 | Japan | G1d |
| 4143 | KR095279 | CH/HNQX-3/14 | 2016 | China | G2d |
| 4143 | MN721363 | CH-GS-TS01 | 2019 | China | G2d |
| 4146 | MH593896 | CH/TP/E5/2018 | 2019 | China | G1d |
| 4146 | JN315706 | CH/FJND-2/2011 | 2011 | China | null |
| 4146 | MN315264 | AH-2018-HF1 | 2020 | China | G1b |
| 4149 | JX018180 | CH/YNKM/2012 | 2012 | China | G1b |
| 4149 | JQ239431 | CH3 | 2012 | China | G1b |
| 4149 | KC109141 | JS2008 | 2013 | China | G1b |
| 4149 | KC210146 | JS2008 | 2013 | China | G1b |
| 4149 | OL870434 | CH/HB/CZ02 | 2022 | China | G2c |
| 4149 | ON058991 | SCMYSWUN03 | 2023 | China | G2c |
| 4149 | MT031818 | CH/QD/2018 | 2020 | China | G1b |
| 4149 | MZ161081 | CH/SCST/04/2020 | 2021 | China | G1d |
| 4149 | KP399615 | CH-WTC1-02-2013 | 2015 | China | G1c |
| 4149 | MN368718 | GDjm18-2 | 2020 | China | G1b |
| 4149 | KY775055 | HBYC1 | 2017 | China | G1d |
| 4149 | MF038016 | PED-JS-2015-12-1 | 2018 | China | G2b |
| 4149 | MK820042 | swun-H3-CH-SCYA-2019 | 2019 | China | G1d |
| 4149 | MH991855 | V7-HB2018 | 2019 | China | G1d |
| 4149 | MG334006 | YnP5 | 2017 | China | G1d |
| 4152 | AF353511 | CV777 | 2001 | Switzerland | G1a |
| 4152 | KJ399978 | OH851 | 2014 | China | G1c |
| 4152 | KR296677 | CH-XBC-01-2015 | 2015 | China | G1c |
| 4152 | MZ161083 | CH/HBTS/09/2020 | 2021 | China | G2c |
| 4152 | MZ161041 | CH/JXPY/07/2020 | 2021 | China | G2a |
| 4152 | MN617863 | CH/SCGY-1/2018 | 2019 | China | G3 |
| 4152 | MW145531 | CH/SCYB-2/2019 | 2022 | China | G1b |
| 4152 | KU133262 | CH-SDRZ-2013 | 2016 | China | G1c |
| 4152 | MN368701 | GDjm17-1 | 2020 | China | G1c |
| 4152 | ON263446 | GNU-2103 | 2022 | South Korea | G2b |
| 4152 | LT906620 | CV777 | 2018 | Germany | G1a |
| 4152 | OP186916 | GNU-2253 | 2022 | South Korea | G2b |
| 4152 | KY775054 | HBEZ3 | 2017 | China | G1c |
| 4152 | JN547228 | CH/S | 2011 | China | G1b |
| 4152 | MW915437 | HT/2017 P150 | 2022 | China | G2a |
| 4152 | KF898124 | KPEDV-9 | 2014 | South Korea | G1a |
| 4152 | MK648473 | NL/GD006/2018 | 2019 | Netherlands | G1c |
| 4152 | LC113937 | OITA2014-5/14 | 2016 | Japan | G1c |
| 4152 | MF038017 | PED-JS-2016-03 | 2018 | China | G1c |
| 4152 | MH991856 | W3-AH2018 | 2019 | China | G2a |
| 4152 | MN692769 | PEDV-1613 | 2020 | Spain | G1c |
| 4152 | MN893415 | CH-HNPDS-2-2018 | 2020 | China | G1c |
| 4152 | KM225252 | YN4-144 | 2016 | China | G2a |
| 4155 | KM225240 | FJ64 | 2016 | China | G2b |
| 4155 | OQ349215 | FJ2006 | 2023 | China | G2b |
| 4155 | MN368724 | GDzj18-1 | 2020 | China | G2b |
| 4155 | KM225244 | HN40 | 2016 | China | G2b |
| 4155 | KT313038 | PEDV4-S-3 | 2015 | China | G2b |
| 4155 | MH991864 | Y9-SX2018 | 2019 | China | G2b |
| 4155 | OP186873 | GNU-2201 | 2022 | South Korea | G2b |
| 4155 | OQ513993 | G2 | 2023 | Vietnam | G2b |
| 4155 | KR941552 | VN2-0514/2014 | 2018 | Vietnam | G2a |
| 4158 | MK532999 | M12-SX2017 | 2019 | China | G2a |
| 4158 | KY619768 | 13JM-291 | 2018 | Japan | G2a |
| 4158 | OL870435 | CH/HB/ZJK01 | 2022 | China | G2c |
| 4158 | KU975416 | CH-SCNC-3-2015 | 2017 | China | G2a |
| 4158 | KJ646582 | FJ-FQ2 2012 | 2014 | China | G2b |
| 4158 | OP235511 | FJLY03-2022 | 2023 | China | G2a |
| 4158 | KF601200 | GDEP/2013 | 2013 | China | G2b |
| 4158 | KF601199 | GXHZ/2013 | 2013 | China | G2a |
| 4158 | KF601201 | GXNN/2013 | 2013 | China | G2b |
| 4158 | KF601198 | JXJA/2013 | 2013 | China | G2b |
| 4158 | MH003891 | HM2017 | 2018 | China | G2a |
| 4158 | KX982554 | VN02/HY/2013 | 2017 | Japan | G2a |
| 4158 | OK642747 | XJ1904-34 | 2021 | China | G2a |
| 4158 | JX647847 | GD-1 | 2012 | China | G2a |
| 4161 | KF468753 | IA1 | 2013 | USA | G2b |
| 4161 | KR078299 | PC21A | 2015 | USA | G2a |
| 4161 | MK532989 | B9-GD2017 | 2019 | China | G2c |
| 4161 | MZ161044 | CH/AHMJ/07/2020 | 2021 | China | G2b |
| 4161 | OL657165 | CH/GX1/F-1/2020 | 2022 | China | G2c |
| 4161 | MZ570141 | CH/HNKF-02/2020 | 2022 | China | G2c |
| 4161 | MN368710 | FJfz18-1 | 2020 | China | G2b |
| 4161 | MW478772 | GDsg13 | 2021 | China | G2c |
| 4161 | KT323980 | LNCT2 | 2016 | China | G2b |
| 4161 | MN893411 | CH-HNLH-2016 | 2020 | China | G2c |
| 4161 | KY793536 | CH/GX/2015/750A | 2017 | China | G2c |
| 4161 | KX073610 | GZMR | 2016 | China | G2b |
| 4161 | ON058990 | SCLSSWUN02 | 2023 | China | G2c |
| 4161 | MK533010 | V15-HB2018 | 2019 | China | G2c |
| 4161 | MW826598 | XT/2017 | 2022 | China | G2c |
| 4164 | KY619779 | 96-P4C6 | 2018 | Japan | G3 |
| 4164 | MN091362 | QRO/LI-DMZC45/2016 | 2020 | Mexico | G2b |
| 4164 | MZ161054 | CH/FJDH/04/2020 | 2021 | China | G2c |
| 4164 | MZ161024 | CH/HBBX/10/2020 | 2021 | China | G2a |
| 4164 | OL870433 | CH/HB/CZ01 | 2022 | China | G2c |
| 4164 | OQ718904 | CH-HeN24-2023 | 2023 | China | G2c |
| 4164 | MW478768 | GDsg09 | 2021 | China | G2c |
| 4164 | ON263438 | GNU-2035 | 2022 | South Korea | G2b |
| 4164 | AB548622 | KH | 2013 | Japan | G3 |
| 4167 | KY211062 | XY6S | 2017 | China | G2b |
| 4167 | KP870139 | CH/GD-29/2014 | 2015 | China | G2a |
| 4167 | MZ161023 | CH/GXDX/10/2020 | 2021 | China | G2a |
| 4167 | OQ349203 | FJ1207 | 2023 | China | G2b |
| 4170 | MG132636 | HB16022 | 2018 | China | G2b |
| 4170 | JQ638918 | CH/HBBD/2011 | 2012 | China | G2a |
| 4170 | MZ161059 | CH/HNLY/03/2020 | 2018 | China | G2a |
| 4170 | MK685665 | CH-SCNJ-2019 | 2019 | China | G2a |
| 4170 | KY828998 | PEDV/MEX/JAL/03/2016 | 2018 | Mexico | G2b |
| 4173 | MZ090589 | C9822-GEN-0315KS2A | 2022 | Thailand | G2a |
| 4173 | OQ349208 | FJ1516 | 2023 | China | G2c |
| 4173 | MK507906 | KNU-1828 | 2019 | South Korea | G2b |
| 4176 | AB548623 | NK | 2013 | Japan | G3 |
| 4176 | MZ161008 | CH/GDMM/12/2020 | 2021 | China | G2c |
| 4176 | KP399608 | CH-LNC-10-2014 | 2015 | China | G2b |
| 4176 | KP399630 | CH-LNC-12-2012 | 2015 | China | G2b |
| 4176 | ON263439 | GNU-2036 | 2022 | South Korea | G2b |
| 4176 | ON263440 | GNU-2037 | 2022 | South Korea | G2b |
| 4179 | KY211046 | NY5S | 2017 | China | G2b |
| 4182 | MK111633 | CH/HNAY/2016 | 2018 | China | G2c |
| 4182 | KX982576 | VN-TH15/HY/2015 | 2017 | Japan | G2a |

Note: null, instead the sequence was not used to divided subgroups.
Abbreviations: No., number; nt, nucleotide.
