# Supplementary material for: The Emergence of Novel Variants of the Porcine Epidemic Diarrhea Virus Spike Gene from 2011 to 2023
Source: Transbound Emerg Dis. 2024 Jul 16;2024:2876278. doi: 10.1155/2024/2876278 (PMC12017180; doi:10.1155/2024/2876278)
Supplement: Supplementary 2 — Table 2: deletions and insertions of the sequence in the G1 group compared with the AF353511 (CV777) sequence. [file 2876278.f2.docx]

**Table S2. Deletions and insertions of the sequence in the G1 group compared with the AF353511 (CV777) sequence.**

| GenBank No. | Deletion site and numbers | Insertion site and numbers |
| --- | --- | --- |
| KJ857455  LT897799 | 4129 to 4149 nt (1377-1383 aa), 21 |  |
| OQ291158 | 4129 to 4149 nt (1377-1383 aa), 21 | 331 to 332 nt (111 aa, corresponding part in G2 is 115 aa), 12 |
| MK820042  MZ16108 | 400 to 402 nt (134 aa, corresponding part in G2 is 140 aa), 3 |  |
| MH593896 | 400 to 402 nt, 3578 to 3580 nt (134 aa, 1193-1194 aa, corresponding part in G2 is 140 aa, 1196-1197aa, respectively), 3, 3 |  |
| KX982564 | 329 to 331 nt, 401 to 403 nt, 3578 to 3580 nt ((110-111 aa, 134-135 aa, 1193-1194 aa, corresponding part in G2 is 114-115aa, 140-141aa, 1196-1197 aa, respectively), 3, 3,3 |  |
| KY775055,  MG334006,  MH991855 | 3578 to 3580 nt (1193-1194 aa) (corresponding part in G2 is 1196-1197 aa), 3 |  |
| KP399615 | 1068 to 1108 nt, 3nt |  |
| MN315264 | 455 to 457 nt, 2323 to 2325 nt (152-153 aa, 775-776 aa, corresponding part in G2 is 157-158 aa, 778-779 aa, respectively), 3,3 |  |
| JQ239431  JX018180  KC109141  KC210146  MN368718  MT031818 | 455 to 457 nt (152-153 aa, corresponding part in G2 is 157-158),3 |  |

Abbreviations: No., number; nt, nucleotide; aa, amino acid.
