# Supplementary material for: The Emergence of Novel Variants of the Porcine Epidemic Diarrhea Virus Spike Gene from 2011 to 2023
Source: Transbound Emerg Dis. 2024 Jul 16;2024:2876278. doi: 10.1155/2024/2876278 (PMC12017180; doi:10.1155/2024/2876278)
Supplement: Supplementary 3 — Table 3: deletions and insertions of the sequence in the G2 group and the G3 group compared with the KT323980 (LNCT2) sequence. (Additional supporting information can be found online in the Supplementary Materials section at the end of this article.) [file 2876278.f3.docx]

**Table S3. Deletions and insertions of the sequence in the G2 group and the G3 group compared with the KT323980 (LNCT2) sequence.**

| GenBank No. | Deletion site and numbers | Insertion site and numbers |
| --- | --- | --- |
| MW915432 | 164 to 169 nt, 4132 to 4158 nt (55-57 aa, 1378 aa-1386 aa), 6, 27 |  |
| MF038016 | 342 to 353 nt (114-118 aa), 12 |  |
| ON263446 | 1127 to 1135 nt (376-379 aa), 9 |  |
| KT313038 | 1136 to 1141 nt (379-381 aa), 6 |  |
| MH991864, OQ513993, KM225240, KM225244 | 164 to 169 nt (55-57 aa), 6 |  |
| OP186916 | 181 to 186 nt (61-62 aa), 6 | 415 to 416 nt (139 aa), 3 |
| OP186873 | 181 to 186 nt (61-62 aa), 6 |  |
| KF601200 KF601201 | 1317 to 1319 nt (439-440 aa), 3 |  |
| ON263439 ON263440 |  | 190 to 191 nt (64 aa), 15 |
| KP399608  KP399630 |  | 1128 to 1129 nt (377 aa),15 |
| ON263438 |  | 1138 to 1139 nt (380 aa), 3 |
| MN091362 |  | 1145 to 1146 nt (382 aa), 3 |
| KY211062 OQ349203 |  | 1150 to 1151 nt (384 aa), 6 |
| KY828998 |  | 692 to 693 nt (231 aa), 9 |
| MK507906 |  | 692 to 693 nt (231 aa), 12 |
| MG132636 |  | 700 to 701 nt (237 aa), 9 |
| KM406180 | 4048 to 4083 nt (1383-1395 aa), 36 |  |
| MW915434 | 164 to 169 nt, 4138 to 4158 nt (55-57 aa, 1380-1386 aa), 6, 21 |  |
| OL870434 | 2972 to 3027 nt, 9 nt deletion  3942 to 4071 nt, 3 nt deletion in three sites |  |
| ON058991 | 164 to 185 nt (55-62 aa), 12 |  |
| MZ161083 | 164 to 185 nt (55-62 aa), 9 |  |
| MN368724 | 177 to 182 nt (59-61 aa), 6 |  |
| OL870435 | 3589 to 3591 nt (1197 aa), 3 |  |
| MW478768 |  | 1663 to 1664 nt (555 aa), 3 |
| MZ161054 |  | 2310 to 2311 nt (771 aa), 3 |
| OL870433 |  | 940 to 970 nt (314 nt), 6 |
| OQ718904 |  | 1175 to 1176 nt (392 aa), 3 |
| OQ349208 |  | 694 to 695 nt (232 aa),12 |
| MZ161008 |  | 1078 to 1179 nt (393 aa),15 |
| KY211046 |  | 1066 to 1067 nt (356 aa), 18 |
| MK111633 |  | 1072 to 1073 nt (358 aa), 21 |
| KM225252 | 430 to 435 nt, 3589-3591 nt (144-145 aa, 1197 aa), 6, 3 |  |
| MH991856 MZ161041 | 164 to 169 nt, 3589-3591 nt (55-57 aa, 1197 aa), 6, 3 |  |
| MW915437 | 182 to 187 nt, 3589-3591 nt (61-63 aa, 1197 aa), 6, 3 |  |
| KR941552 | 2237 to 2239 nt, 3589-3591 nt (746-747 aa, 1197 aa), 3, 3 |  |
| KF601199 | 3489 to 3491 nt (1163-1164 aa), 3  one nt insertion at 1190 site and one nt deletion at 1214 site |  |
| MZ161024 | 3589 to 3591 nt (1197 aa), 3 | 3811 to 3812 nt (1271 aa), 6 |
| MZ161023 | 3589 to 3591 nt (1197 aa), 3 | 3811 to 3812 nt (1271 aa), 9 |
| JQ 638918 |  | 693 to 694 (231-232 aa), 12 |
| KP870139 | 3589 to 3591 nt (1197 aa), 3 | 4138 to 4139 nt (1380), 9 |
| MK685665 | 3589 to 3591 nt (1197 aa), 3 | 1125 to 1126 nt (376 aa), 12 |
| MZ161059 | 3589 to 3591 nt (1197 aa), 3 | 3811 to 3812 nt (1271 aa),12 |
| MZ090589 |  | 699 to 700 nt (234 aa), 12 |
| KX982576 | 3589 to 3591 nt (1197 aa), 3 | 1148 to 1149 nt (383 aa), 24 |
| KU975416  KX982554  KY619768  MH003891  MK532999  OK642747  OP235511 | 3589 to 3591 nt (1197 aa), 3 |  |
| KR095279  MN721363 | 175 to 186 nt, 209 to 211 nt, 420 to 422 nt (59-62 aa, 70-71 aa, 140-141 aa), 12, 3, 3 |  |
| KP890336  MN721362  MN721368 | 175 to 186 nt, 209 to 211 nt, 417 to 422 nt (59-62 aa, 70-71 aa, 139-141 aa), 12, 3, 6 |  |
| MN617863 | 167 to 191, 416 to 418 (56-64 aa,139-140 aa)  12, 3 | 479 to 480 nt (160 aa), 6 |
| AB548622, KY619779, |  | 191 to 192 nt (64 aa), 3 |
| AB548623 |  | 191 to 192 nt, 1829 to 1830 nt (64 aa, 610 aa), 3,12 |

Abbreviations: No., number; nt, nucleotide; aa, amino acid.
